# Crystallized Hopeine

**Published:** 1886-04

**Authors:** 


### Medical News.

### Crystallized Hopeine.

Dujardin-Beaumetz, in the *Bulletin Gen. de Therapeutique*, shows conclusively that this substance, sold by the "Concentrated Produce Company," of London, for fabulous prices, is nothing but morphine, flavored with hops. It was discovered that this hopeine of Williamson had the same chemical, polariscopic, microscopic and physiological characteristics as morphine, and it was therefore safe to assume that it was morphine. Dujardin-Beaumetz furthermore claims, that Roberts, of New York, and Smith, of England, both of whom have experimented with this supposed hopeine, and have published the results of their investigations, were really using morphine; and peculiar actions of the drug, viz., absence of headache after sleep, etc., were entirely accidental. The only genuine hopeine is a brown, resinous substance, not alcaloidal in its nature, having slight hypnotic qualities.

